# Sero‐prevalence and risk factors associated with brucellosis in dairy cattle of Sylhet District, Bangladesh: A cross‐sectional study

**DOI:** 10.1002/vms3.1100

**Published:** 2023-03-03

**Authors:** Nirmalendu Deb Nath, Syed Sayeem Uddin Ahmed, Vashkar Malakar, Tanimul Hussain, Liton Chandra Deb, Suman Paul

**Affiliations:** ^1^ Department of Epidemiology and Public Health Faculty of Veterinary, Animal and Biomedical Sciences, Sylhet Agricultural University Sylhet Bangladesh; ^2^ Department of Biomedical and Diagnostic Sciences College of Veterinary Medicine, University of Tennessee Knoxville Tennessee USA; ^3^ Department of Population Health and Pathobiology College of Veterinary Medicine, North Carolina State University Raleigh North Carolina USA

**Keywords:** brucellosis, prevalence, risk factors, rose bengal test (RBT), serum agglutination test (SAT)

## Abstract

**Background:**

Brucellosis is an emerging disease that causes a significant impact on productive and reproductive performance in dairy cattle. Though *Brucella* is a pivotal microorganism for dairy cattle, the scenario of brucellosis in Sylhet District is unknown.

**Objectives:**

A cross‐sectional study was carried out to assess the prevalence and determinants associated with brucellosis in dairy cattle of Sylhet District.

**Methods:**

A total of 386 sera and data on determinants from 63 dairy herds were collected from 12 sub‐districts using simple random sampling. The sera were tested with Rose Bengal *Brucella* antigen test, *Brucella* abortus plate agglutination test and serum agglutination test to find out the sero‐positivity.

**Results:**

Overall, 17.09% (95% CI: 13.67–21.18) prevalence in cows were calculated. Relatively higher prevalence (56.08%; 95% CI: 42.23–70.32) was recorded in cows having parity ≥4 and were at higher risk (OR = 7.28) than the other cows with parity 0–3. Prevalence was significantly higher in cows with history of abortion 90.63% (95% CI: 75.79–96.76), repeat breeding 79.17% (95% CI: 65.74–88.27) and reproductive abnormalities 48.54% (95% CI: 39.12–58.07). Farm‐level prevalence was high in farms with the previous history of abortion 95.45% (95% CI: 78.20–99.19) and repeat breeding 90.00% (95% CI: 74.38–96.54).

**Conclusions:**

The prevalence was high in Sylhet district, which might be a public health concern. Therefore, this study would represent the baseline information for guiding brucellosis control and prevention.

## Graphical abstract

Brucellosis is an emerging disease, which is highly prevalent in Sylhet district of Bangladesh. Prevalence was significantly higher in cows with history of abortion, repeat breeding and reproductive abnormalities, which might be a public health concern.

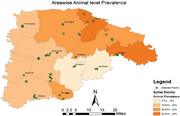

## 1 INTRODUCTION

Bovine brucellosis is caused by gram‐negative, non‐spore‐forming, non‐motile and non‐capsulated coccobacilli bacteria. Brucellosis is an important zoonotic disease (Corbel, 2006; von Bargen et al., 2012) and is responsible for public health and economic impacts in endemic areas (Preis et al., 2019; Scacchia et al., 2013). In cattle, the disease is generally brought about by ingesting contaminated feed or water with secretion, or aborted foetal remains from infected cows or by licking the vaginal discharges and genitals of aborted foetuses (Díaz, 2013). Brucellosis may cause 30%–70% abortions in the last trimester in fully susceptible herds (Godfroid et al., 2005). Infection may last long in cows during subsequent pregnancies; the recurrence of abortion is rare, but uterine and mammary infections commonly recur (Pappas et al., 2005). It also prolongs inter‐calving intervals and reduces calves’ lifetime production. Thus, the economic impact of the disease at the national level is substantial (Radostits et al., 2006).

Some countries successfully controlled or eradicated bovine brucellosis through test and slaughter programmes (Godfroid & Käsbohrer, 2002; Ragan, 2002). However, the disease still occurs at varying prevalences, particularly in developing countries, including Bangladesh (Kadohira et al., 1997; Omer et al., 2000; Moreno, 2002; Poester et al., 2002; Hegazy et al., 2011; Rahman, 2015; Logonda et al., 2022). The countries at high risk of brucellosis are countries in the Mediterranean Sea Basin (Portugal, Spain, South France, Italy, Greece, Turkey, Algeria, Tunisia, Egypt and Morocco), likewise nations of South and Central America, Asia, Africa, the Caribbean and the Middle East. The most prevalent endemic countries of bovine brucellosis are Libya, Georgia, Algeria, Nigeria, Zambia, Turkey, India, Pakistan and so on (Minas, 2006; Pappas et al., 2006; Galinska & Zagórski, 2013).

The prevalence of bovine brucellosis varies widely across Bangladesh, with a reported sero‐prevalence of 0.3%–20.5% (Rahman et al., 2011; Rahman, 2015). The prevalence of brucellosis of cattle in Mymensingh, Central Cattle Breeding and Dairy Farm, Dhaka and Sirajganj was estimated at 0.3%, 20.5% and 2.66%, respectively (Rahman et al., 2011; Rahman, 2015). Many cases of abortion, retention of placenta, metritis and stillbirth, which may be due to brucellosis, remain undiagnosed and untreated in Bangladesh (Rahman et al., 2010). As a result, the chance of having brucellosis has increased tremendously. Prevalence studies in other surrounding countries indicated 12%–20% in India (Deka et al., 2018), 3%–8.7% in Pakistan (Shafee et al., 2011; Arif et al., 2019) and 13.4% in Nepal (Pandeya et al., 2013). Brucellosis has also been reported in many other parts of Asia (Bhatia & Narain, 2010), although detailed information on its prevalence is still lacking in most countries.

The risk factors responsible for the occurrence of bovine brucellosis include the purchase of infected cattle for replacement, the nature of animal production, demographic factors, regulatory issues, climate and wildlife interaction (Muma et al., 2007; ZareBidaki et al., 2022). Furthermore, one major contributing factor to the spreading of the disease is free movement of animals (Ocholi et al., 2004). In addition, management systems, herding of different species together, use of common pastures and water sources, age, breed, sex, lactation status and season are some other factors that may influence the prevalence of brucellosis (Junaidu et al., 2010; Bertu et al., 2012; Boukary et al., 2013).

Sylhet is a hilly and hoar (wetland ecosystem) based district situated in the northeastern part of Bangladesh, and several sub‐districts share borders with the neighbouring country India. Many legal and illegal trades of cattle occur between India and Bangladesh through cross‐border cattle movements in this area. This may increase the risk of brucellosis in cattle as it is a transboundary animal disease. Most of the farms in Sylhet region experienced a lot of abortion and stillbirth, which might be due to brucellosis. Unfortunately, information regarding the sero‐prevalence and determinants of bovine brucellosis in the Sylhet district has remained unknown. Therefore, the present study aimed to explore the burden and identify the determinants of bovine brucellosis in the Sylhet district.

## 2 MATERIALS AND METHODS

### 2.1 Study area

The study area includes all 12 sub‐districts in Sylhet district, which are located at 24°36′–25°11′ north latitudes and 91°38′—92°30′ east longitudes, occupying an area of 3452.07 km^2^. The district is situated in the region with hills and basins, which constitute one of the most distinctive regions in Bangladesh. Sylhet is bordered by India's Meghalaya, Assam and Tripura states in the east and south, respectively.

### 2.2 Sample size determination

The target population includes local indigenous and crossbred cows from all 12 sub‐districts of Sylhet district. The sample size was calculated by using the formula for estimating prevalence according to Thursfield (2005).

$$\frac{Z^{2}\times \;P\;\times \;\left(1-P\right)}{d^{2}}$$

As no reliable prior information on the prevalence of bovine brucellosis in Sylhet was available, an expected prevalence (Pexp) of 50% was used to maximize the sample size. Using this Pexp with a desired absolute precision *d* = 0.05 and *Z* = 1.96 for a 95% confidence interval, a required sample size of at least 384 was determined.

### 2.3 Study design

A cross‐sectional study was conducted for 6 months in the study area. Lists of the farms were collected from the local sub‐district livestock offices. A minimum of five farms from each sub‐district and six cows from each farm were selected using a simple random sampling technique. However, in some sub‐districts, it was difficult to select 5 farms to fulfil the inclusion criteria, and finally, 63 farms from 12 sub‐districts were included in the study. Then, blood samples from the lactating cows of selected farms were collected to achieve the desired number of samples size. Finally, 386 serum samples from 63 selected farms were processed from the blood samples for this study.

### 2.4 Serum tested by buffered *Brucella* antigen tests (BBATs)

The protocols used for two BBATs; Rose Bengal Test (RBT) and Plate Agglutination Test for *Brucella* abortus (BPAT) were essentially the same described by Alton et al. (1988). First, the preserved serum samples were brought to room temperature from −20°C and allowed to thaw. After thawing, an equal volume (30 μL) of serum and antigen (containing Bacterial suspension of *Brucella* organisms stained with Rose Bengal dye and buffered at pH 3.6 and 0.95 g/L of sodium aside) was taken in a glass slide. Then, by a small glass rod, the mixture of serum and antigen was allowed to rotate for 4 min. The result was considered positive when any agglutination was observed within the required period.

### 2.5 Serum agglutination test (SAT)

Serum agglutination test (SAT) was another serological test used to detect the antibody from the serum of *Brucella* infected animals, which was described by Rahman (2015) and Rahman et al. (2013). Briefly, 168 μL of Serum Agglutination de Wright (SAW) buffer in the 1st well, and 100 μL were added to the 2nd and 3rd well of 96 well plates. Then 32 μL of preserved serum samples were added to the 1st well (dilution 1/6.25). After mixing, transferred 100 μL from the 1st to 2nd well (dilution 1/12.5) and followed by the 3rd (dilution 1/25) and finally discarded 100 μL from 3rd well. Each serum sample was duplicated, and a total of 15 serum samples were loaded in the 96 wells microtitre plate. Next, 100 μL of standardized SAW antigen was added to each well giving the serial dilution of 1/12.5, 1/25 and 1/50. Then the plates were incubated at 37°C for 20–24 h. Reading was done based on agglutination and expressed as International Unit (IU). A sample was considered to be *Brucella*‐positive if it contained ≥30 IU/mL.

### 2.6 Case definition

If a cow's serum sample showed a positive reaction in any of the three tests (RBT, BPAT, SAT), the cow was identified as positive for bovine brucellosis. On the other hand, if the serum sample of a cow has shown negative results in all three tests, then that cow was considered negative. However, in the case of farm level, if at least one cow from a particular farm was classified positive for bovine brucellosis, that farm was selected as bovine brucellosis‐positive.

### 2.7 Data collection and management

The owners of the selected farms were interviewed face to face using a well‐designed questionnaire to record the farm and cow‐level information. The questionnaire included different types of farm and individual animal‐level information related to risk factors. Type of house barn, sanitary condition, replacement stock, history of abortion, abnormal uterine discharge, retention of placenta, problem in heat and problem‐related to repeat breeding were associated with brucellosis were captured for both farm‐ and animal‐level questionnaires to find out the relationship with the sero‐positivity of brucellosis. Then collected individual level, farm level and laboratory information was initially entered into a Microsoft Excel spreadsheet and coded for analysis. Preliminary descriptive analysis was done with 10 variables, including one pseudo‐continuous variable (herd size) and nine categorical variables for estimating prevalence and identifying the determinants of bovine brucellosis. Herd size was categorized into two levels (≤20 and >20) categorical variable by taking the mean of the herd size as cut‐off to make a homogenous distribution in each category. Furthermore, different stages of pregnant animals were found among the lactating cows and categorized as parity 1–4 (when the animals were in parity ≥4 were considered parity number four).

### 2.8 Statistical analysis

The results obtained from the cross‐sectional study were used for prevalence and risk factors analysis. Prevalence was calculated as the proportion of positive cows/farms among the sampled cows/farms. The precessions of these estimates were ensured by calculating the 95% confidence interval of the proportions. Differences in prevalence between the categories of cows/farms level traits were compared using chi‐square (*χ* ^2^) test. The geographical distribution and choropleth maps of individual animal and farm level prevalence of bovine brucellosis were generated in ArcGIS 10.1 (Chandra Deb et al., 2022). Then, a case–control study was designed based on *Brucella*‐positive or negative cows to assess the determinants of bovine brucellosis in selected cows and farms. For the case–control analysis, a 1:1 case and control ratio was used, where for each case, a control was randomly selected from completely brucellosis‐free cows or farms. Logistic regression analysis was carried out to explore the association between independent variables with the dichotomous dependent variable. Correlation among the explanatory variables was checked for multicollinearity as correlation coefficient (*r* ^2^) ≥0.5 was used as a cut‐off. Chi‐square (*χ*2) tests and odds ratios were applied to test relationships between *Brucella‐*positive cows with ordinal and dichotomized independent variables. Explanatory variables with *p*‐values ≤0.20 in univariable analyses were used in the multivariable logistic regression analysis. In multivariable analysis, a backward elimination procedure was used. The confounding effect of two explanatory variables was also evaluated by assigning the change of parameter estimates before and after removing a variable from the model. If the parameter estimate of a variable increased or decreased ≥20% after removing another variable from the model, these two explanatory variables were considered to have a confounding effect on the outcome variable. Finally, a critical *p*‐value ≤0.05 was considered statistically significant to identify the determinants of bovine brucellosis.

## 3 RESULTS

### 3.1 Prevalence and distribution of bovine brucellosis in dairy animals

**FIGURE 1:**
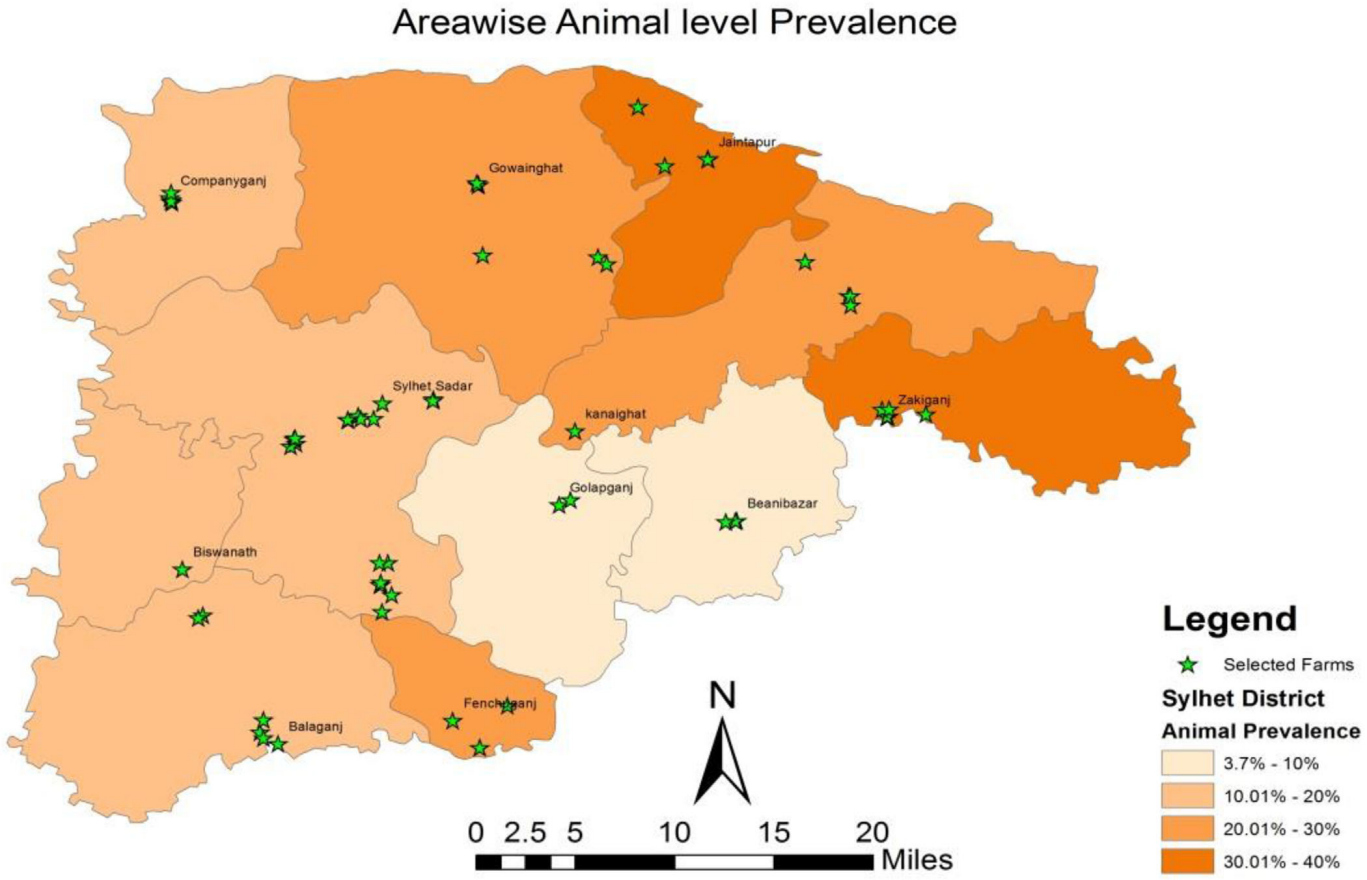
Prevalence map of bovine brucellosis at cow level in Sylhet district estimated in a cross‐sectional study.

Out of 386 individual serum samples, 36 (9.33%), 46 (11.92%) and 58 (15.03%) were identified positive for bovine brucellosis in RBT, SAT and BPAT, respectively. A total 66 (17.09%) serum samples showed positive response against the *Brucella* antigen, and therefore, the prevalence was estimated as 17.09% (95% CI: 13.67%–21.18%) (Table 1). There was no significant differences in prevalences (crossbreed 17.12% and local breed 17.07%) between breeds. However, the prevalence increased with the increasing number of parity, where the highest prevalence was found in parity ≥4 (56.82%; 95% CI: 42.23%–70.32%). Prevalence was significantly high in cows with a history of abortion and repeat breeding. Moreover, the prevalence of bovine brucellosis in cows differed significantly among sub‐districts, and the highest prevalence of brucellosis was recorded in Jaintapur (36.00%; 95% CI: 23.63%–52.18%) and the lowest in Golapganj (3.70%; 95% CI: 1.45%–6.22%) sub‐district (Figure 1).

**TABLE 1:** Prevalence of bovine brucellosis in cows estimated from 386 randomly collected serum samples from 63 cattle farms of 12 sub‐districts.

Variables	No of cow tested (%)	No of cow positive (%)	Prevalence (%) (95% confidence interval)	*p*‐Value
Overall	386 (100.00)	66 (100.00)	17.09 (13.67–21.18)	
Breed				0.99
Cross	222 (57.51)	38 (57.58)	17.12 (12.73‐22.62)	
Local	164 (42.49)	28 (42.43)	17.07 (12.08‐23.57)	
Parity				<0.01
0	28 (7.25)	0 (0)	0	
1	157 40.67)	10 (15.15)	6.37 (3.5–11.33)	
2	116 (30.05)	16 (24.24)	13.79 (8.67–21.23)	
3	41 (10.62)	15 (22.730	36.59 (23.59–51.88)	
≥4	44 (10.62)	25 (37.88)	56.8 (42.23–70.32)	
Cows with abortion history				<0.01
Yes	32 (8.29)	29 (43.94)	90.63 (75.79–96.76)	
No	354 (91.70)	37 (56.06)	10.54 (7.68–14.07)	
Cows with repeat breeding history				0.02
Yes	48 (12.44)	38 (57.58)	79.17 (65.74–88.27)	
No	338 (87.56)	28 (42.42)	8.28 (5.79–11.71)	
Cows with history of other reproductive abnormalities				<0.01
Yes	103 (26.68)	50 (75.760	48.54 (39.12–58.07)	
No	283 (73.32)	16 (24.24)	5.65 (3.51–8.980	
Herd size				0.97
Large	128 (33.16)	22 (33.33)	17.17 (11.64–24.66)	
Small	258 (66.84)	44 (66.67)	17.05 (12.95–22.11)	
Insemination practice in farm				0.90
Natural	254 (65.80)	43 (65.15)	16.93 (12.82–22.03)	
Artificial	132 (34.19)	23 (34.85)	17.42 (11.90–24.79)	
Farms’ history of abortion				0.02
Present	133 (34.46)	31 (46.97)	23.31 (16.94–31.18)	
Absent	253 (65.54)	35 (53.03)	13.83 (10.11–18.63)	
Farms’ history of repeat breeding				0.26
Present	198 (51.29)	38 (57.58)	19.19 (14.31–25.24)	
Absent	188 (48.70)	28 (42.42)	14.89 (10.51–20.68)	
Farms’ history of other reproductive abnormalities				0.34
Present	35 (9.070)	8 (12.120)	22.86 (12.07–39.02)	
Absent	253 (65.54)	58 (87.88)	22.92 (18.17–28.42)	

### 3.2 Farm‐level prevalence

**FIGURE 2:**
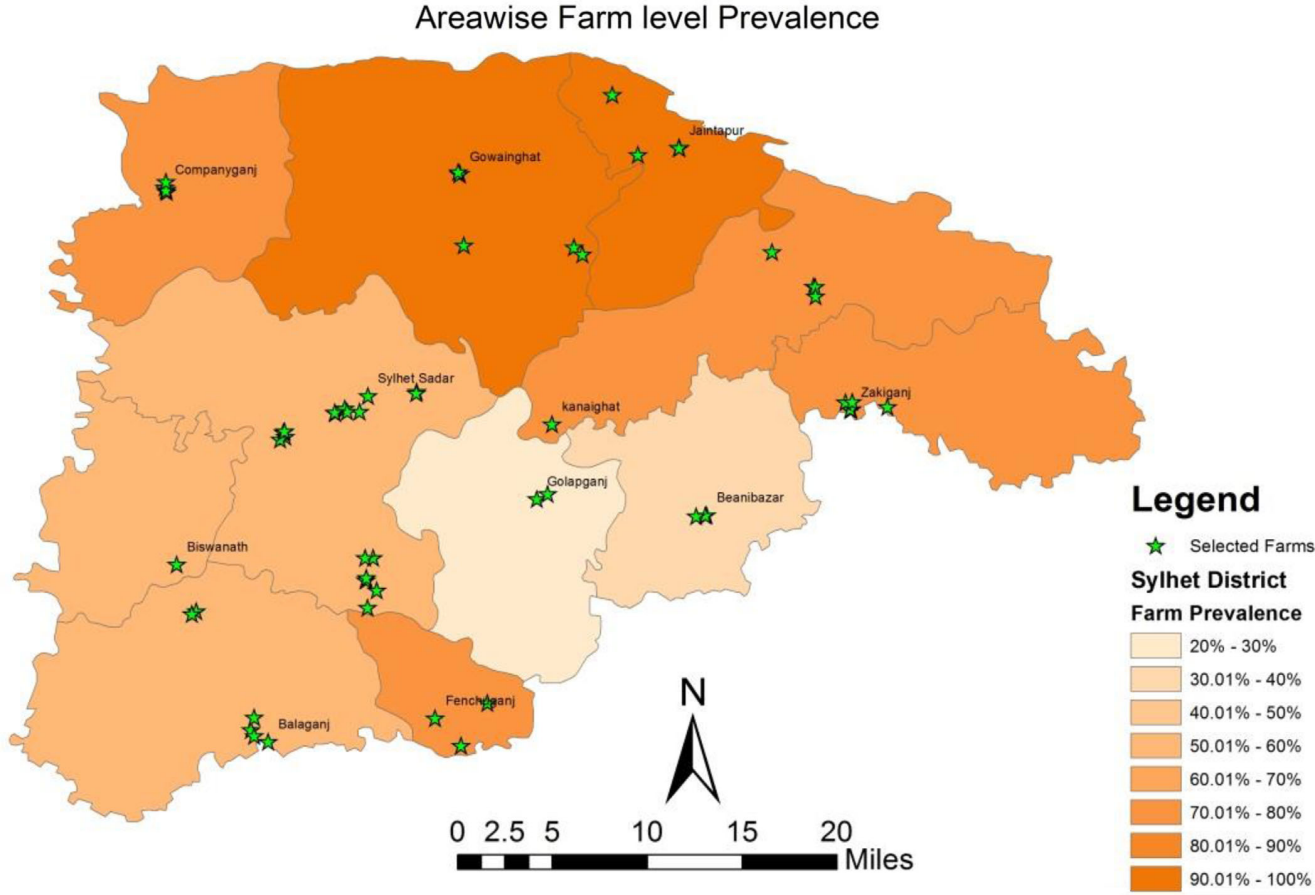
Prevalence map of bovine brucellosis at farm level in Sylhet district estimated in a cross‐sectional study.

A total of 46 farms out of 63 had at least 1 cow showing a positive reaction against *Brucella* antigens and were considered positive farms; therefore, the prevalence of bovine brucellosis at the farm level was 73.02% with 95% CI: 60.97%–82.42%. Farm‐level prevalence was significantly high in farms with the previous history of abortion and repeat breeding. It is worth noting that prevalence was comparatively higher in large farms (88.89%) than in small farms (64.44%). High prevalences at farm level were observed in almost all sub‐districts of Sylhet district, and it ranged from 20% (95% CI: 11.56%–28.32%) to 100% (95% CI: 56.55%–100%). The highest farm prevalence was found in Gowainghat and Jaintapur sub‐districts and the lowest in Golapganj sub‐district (Figure 2).

### 3.3 Determinants at cow level

The univariable association of each determinant with bovine brucellosis among dairy animals is shown in Table 2. The following variables showed potentially significant (*p* ≤ 0.20) associations with brucellosis: parity, cows with abortion history, cows with history of repeat breeding, cows with history of reproductive abnormalities and cows from the farm with a previous record of abortion, repeat breeding and reproductive abnormalities. The results of the final multivariable logistic regression model (Table 3) showed that the significant determinants of bovine brucellosis were parity, animals with the previous history of abortion, repeat breeding and other reproductive abnormalities. The odds of bovine brucellosis among cows with parity ≥4 were 7.82 times higher than the cows with parity one. Similarly, the odds of brucellosis in cows with the previous history of abortion was 5.89 (95% CI: 1.00–34.72) times higher than the cows without history of abortion. In addition, cows with history of repeat breeding (OR = 9.74, 95% CI: 1.78–53.35) and other reproductive abnormalities (OR = 3.10, 95% CI: 1.08–8.90) had higher odds of bovine brucellosis compared to the cows with without previous history of repeat breeding and other reproductive abnormalities, respectively.

**TABLE 2:** Univariate analysis (Chi‐square [*χ*2] test) of plausible determinants of bovine brucellosis in cows from a case–control study with 132 serum samples of cows (66 case and 66 controls).

Variables	Odds ratio (95% confidence interval)	*p*‐Value
Breed		0.86
Local	1.06 (0.53–2.13)	
Cross	1	
Parity		<0.01
≥4	22.49 (6.26–80.84)	
3	7.71 (2.44–24.34)	
2	3.03 (1.14–8.03)	
1	1	
Cows with abortion history		<0.01
Yes	25.08 (5.66–111.17)	
No	1	
Cows with repeat breeding history		<0.01
Yes	43.43 (9.79–192.62)	
No	1	
Cows with history of other reproductive abnormalities		<0.01
Yes	9.00 (4.09–19.81)	
No	1	
Herd size		0.37
Larger	0.72 (0.36–1.47)	
Smaller	1	
Insemination		0.85
Natural	1.06 (0.52–2.20)	
Artificial	1	
Cows with farms’ history of abortion		0.02
Present	2.36 (1.14–4.88)	
Absent	1	
Cows with farms' history of repeat breeding		0.12
Present	1.44 (0.73–2.86)	
Absent	1	
Cows with farms' history of other reproductive abnormalities		0.12
Present	2.30 (0.73–11.44)	
Absent	1	

**TABLE 3:** Final model of multivariate logistic regression analysis of plausible determinants of bovine brucellosis in cows from a case–control study with 132 serum samples of cows (66 cases and 66 controls).

Variables	Odds ratio (95% confidence interval)	*p*‐Value
Parity		0.02
≥4	7.82 (1.70–36.07)	
3	4.81 (1.22–18.96)	
2	1.70 (0.49–5.90)	
1	1	
Cows with abortion history		0.05
Yes	5.89 (1.00–34.72)	
No		
	1	
Cows with repeat breeding history		0.01
Yes	9.74 (1.78–53.35)	
No	1	
Cows with history of other reproductive abnormalities	0.03	
Yes	3.10 (1.08–8.90)	
No	1	

### 3.4 Determinants at the farm level

In the univariable analysis, herd size and farms' history of repeat breeding had a significant association (*p* ≤ 0.20) with the bovine brucellosis‐positive farm. However, in the multivariable logistic regression final model, only farms' history of repeat breeding showed a significant (*p* 0.02) positive association with the brucellosis‐positive farm. The odds of farms with the history of repeat breeding was 6.25 (95% CI: 1.25–31.16) times higher than those without history of repeat breeding.

## 4 DISCUSSION

The study investigated the prevalence and risk factors of bovine brucellosis in Sylhet district of Bangladesh. The burden of bovine brucellosis may vary in the Sylhet region, but little is known about the prevalence, determinants and geographic disparities. Therefore, the findings of the current study are pivotal to identifying the risk factors and sub‐districts with high prevalence so as to provide proper prevention and control measures to combat the bovine brucellosis burden.

### 4.1 Prevalence of bovine brucellosis

This study confirmed a high prevalence of bovine brucellosis in the dairy cows of Sylhet district. Similar to our findings, few other earlier studies in Bangladesh also reported such a high prevalence in individual cattle (Rahman et al., 2011; Rahman, 2015). In addition, the prevalence (20.5%) of brucellosis in Government Central Cattle Breeding and Dairy Farm (CCBDF) in Savar, Dhaka, Bangladesh reported by Rahman (2015) was comparable with the findings of the present study. It is worth noting that Sylhet district is topographically a hoar based area with high rainfall, and most of the grazing lands remain damp for a considerable period of a year which might be a possible reason behind this observed high prevalence. Wetlands near the farm area were identified as a contributing factor to increasing prevalence in a study in Brazil (Borba et al., 2013). Moreover, Sylhet district shares a border with the neighbouring country India. Many legal and illegal cattle trade happen between India and Bangladesh through cross‐border cattle movements. A previous study by Kubuafor et al. (2000) reported that cross‐border cattle movement significantly impacts brucellosis transmission. However, on the other hand, the findings of the present study is in disagreement with Rahman et al. (2006), who reported low animal‐level sero‐prevalence of brucellosis in cattle with 2.4%–8.4%. Another later investigation on bovine brucellosis in the Mymensingh region by Rahman (2015) recorded only 0.3% prevalence in cattle. Geo‐ecological differences along with the difference in study design could be a probable reason behind the differences in prevalence estimates.

### 4.2 Risk factors of bovine brucellosis

This study revealed that the risk of bovine brucellosis in dairy cows increased with increasing age (age was indicated by the number of parity), and the highest odds was observed in cows with ≥4 parity. The association of age with sero‐positivity to brucellosis in cows is also consistent with the findings of previous studies (Al‐Majali et al., 2009; Radostits et al., 2006; Mai et al., 2012; Aulakh et al., 2008). Age is one of the intrinsic factors which influence susceptibility to *Brucella* infection. Brucellosis may increase with age due to prolonged exposure to the pathogen and prolonged antibody response duration in infected animals, particularly in traditional husbandry practice where females are maintained in herds over a long period of time.

In this study, it is found that cows with a history of abortion were significantly associated with *Brucella* sero‐positivity, which is in agreement with other previous studies (Makita et al., 2011; Megersa et al., 2011; Patel et al., 2014). In addition, a previous study conducted by Magona et al. (2009) in Uganda also reported the history of abortion at the herd level as a significant factor for brucellosis. This might be due to the fact that the *Brucella* organism may present in the aborted foetus and play a pivotal role in the transmission of brucellosis through aborted materials and contaminated milk and urine. It is worth noting that the prevalence of brucellosis was comparatively similar in crossbred and local bred cows, and no significant association exists between breeds, as a similar result was documented by Mai et al. (2012). It might be due to the fact that genetic variations between breeds do not significantly influence brucellosis. Moreover, better management practices in the farms might be another possible factor for the low prevalence of brucellosis in both the crossbred and local bred.

It was observed that the previous history of repeat breeding of cow and farms was also significantly associated with bovine brucellosis at animal and farms level, respectively. A study in Gujarat reported that farms with a history of repeat breeding had a higher risk of brucellosis than farms without that history (Patel et al., 2014). This study also identified that cows’ previous history of reproductive abnormalities other than abortion and repeat breeding, like stillbirth, retained placenta and so on increases the risk of brucellosis in individual cows. Moreover, Patel et al. (2014) also described a similar association between brucellosis and the history of repeat breeding and retained placenta, however, at herd level. Therefore, it is worth considering that there is a causal relationship between bovine brucellosis and reproductive abnormalities. However, it needs to be clarified, out of the scope of this study, whether the previous history of reproductive abnormalities increases the risk of cows being *Brucella*‐positive or whether these previous episodes of reproductive abnormalities resulted from exposure to *Brucella* organisms.

## 5 CONCLUSION

The study showed the importance of direct contact with the aborted foetuses, retained placenta and vaginal fluid may be the important source of transmission. Individual cow factors like repeat breeding, abortion, parity and other reproductive abnormalities significantly act as risk factors for brucellosis. Thus, the findings of this study could be used as tools for adoptive surveillance and effective control and prevention of the disease. Further epidemiological investigation on disease transmission, ecological and environmental factors, geographical disparities, economic analysis of the effect of the disease and so on are needed to explore the complete epidemiology and ecology of bovine brucellosis in Sylhet, Bangladesh.

## AUTHOR CONTRIBUTIONS

*Conceptualization; data curation; formal analysis; methodology; software; validation; writing – original draft*: Nirmalendu Deb Nath. *Investigation; software; supervision; validation; visualization; writing review and editing*: Syed Sayeem Uddin Ahmed. *Data curation; investigation; visualization; writing review and editing*: Vashkar Malakar. *Data curation; investigation; writing review and editing*: *Tanimul Hussain*. *Methodology; supervision; validation; visualization; writing review and editing*: Liton Chandra Deb. *Conceptualization; investigation; supervision; validation; writing review and editing*: Suman Paul.

## CONFLICT OF INTEREST STATEMENT

The authors declare that there was no conflict of interests.

## ETHICS STATEMENT

This study was approved by the Sylhet Agricultural University, Bangladesh (SAU/Ethical committee/AUP/18/13), and all study methods were carried out in accordance with relevant guidelines and regulations.

### PEER REVIEW

The peer review history for this article is available at https://publons.com/publon/10.1002/vms3.1100.

## Data Availability

The data that support the findings of this study are available from the Corresponding author upon reasonable request.

## References

[vms31100-bib-0001] Al‐Majali, A. M. , Talafha, A. Q. , Ababneh, M. M. , & Ababneh, M. M. (2009). Seroprevalence and risk factors for bovine brucellosis in Jordan. Journal of Veterinary Science, 100(1), 61–65. 10.4142/jvs.2009.10.1.61 PMC2801095 19255525

[vms31100-bib-0002] Alton, G. G. , Jones, L. M. , Angus, R. , & Verger, J. (1988). Techniques for the brucellosis laboratory. Institut National de la recherche Agronomique (INRA).

[vms31100-bib-0003] Arif, S. , Thomson, P. C. , Hernandez‐Jover, M. , McGill, D. M. , Warriach, H. M. , Hayat, K. , & Heller, J. (2019). Bovine brucellosis in Pakistan; an analysis of engagement with risk factors in smallholder farmer settings. Veterinary Medicine and Science, 5(3), 390–401. 10.1002/vms3.165 30957947 PMC6682800

[vms31100-bib-0004] Aulakh, H. , Patil, P. , Sharma, S. , Kumar, H. , Mahajan, V. , & Sandhu, K. (2008). A study on the epidemiology of bovine brucellosis in Punjab (India) using milk‐ELISA. Acta Veterinaria Brno, 77(3), 393–399. 10.2754/avb200877030393

[vms31100-bib-0005] Bertu, W. J. , Gusi, A. M. , Hassan, M. , Mwankon, E. , Ocholi, R. A. , Ior, D. D. , Husseini, B. A. , Ibrahim, G. , Abdoel, T. H. , & Smits, H. L. (2012). Serological evidence for brucellosis in Bos indicus in Nigeria. Tropical Animal Health and Production, 44(2), 253–258. 10.1007/s11250-011-0011-2 22086409

[vms31100-bib-0006] Bhatia, R. , & Narain, J. P. (2010). Review paper: The challenge of emerging zoonoses in Asia pacific. Asia‐Pacific Academic Consortium for Public Health, 22(4), 388–394. 10.1177/2F1010539510370908 20462853

[vms31100-bib-0007] Borba, M. , Stevenson, M. , Goncalves, V. , Neto, J. F. , Ferreira, F. , Amaku, M. , Telles, E. , Santana, S. , Ferreira, J. , & Lobo, J. (2013). Prevalence and risk‐mapping of bovine brucellosis in Maranhao State, Brazil. Preventive Veterinary Medicine, 110(2), 169–176. 10.1016/j.prevetmed.2012.11.013 23218657

[vms31100-bib-0008] Boukary, A. R. , Saegerman, C. , Abatih, E. , Fretin, D. , Bada, R. A. , De Deken, R. , Harouna, H. A. , Yenikoye, A. , & Thys, E. (2013). Seroprevalence and potential risk factors for *Brucella* spp. Infection in traditional cattle, sheep and goats reared in urban, periurban and rural areas of niger. PLoS One, 8(12), e83175. 10.1371/2Fjournal.pone.0083175 24358261 PMC3865157

[vms31100-bib-1048] Chandra Deb, L. , Ahmed, S. S. U. , Baidhya, C. C. , Deb Nath, N. , Ghosh, S. , & Paul, S. (2022). Prevalence of Eimeria spp. with associated risk factors in dairy calves in Sylhet, Bangladesh. Veterinary Medicine and Science, 8(3), 1250–1257. 10.1002/vms3.776 35202516 PMC9122449

[vms31100-bib-0009] Corbel, M. J. (2006). Brucellosis in humans and animals. World Health Organization, Geneva, Switzerland.

[vms31100-bib-0010] Deka, R. P. , Magnusson, U. , Grace, D. , & Lindahl, J. (2018). Bovine brucellosis: Prevalence, risk factors, economic cost and control options with particular reference to India‐a review. Infection Ecology & Epidemiology, 8(1), 1556548. 10.1080/20008686.2018.1556548

[vms31100-bib-0011] Díaz, A. E. (2013). Epidemiology of brucellosis in domestic animals caused by *Brucella melitensis*, *Brucella suis* and *Brucella abortus* . Revue Scientifique Et Technique (International Office of Epizootics), 32(1), 53–60. 23837364

[vms31100-bib-0012] Galinska, E. M. , & Zagórski, J. (2013). Brucellosis in humans‐etiology, diagnostics, clinical forms. Annals of Agricultural and Environmental Medicine, 20(2), 233–238. Retrieved from http://agro.icm.edu.pl/agro/element/bwmeta1.element.agro‐d734f59d‐dfd9‐44e9‐be70‐7250bede49d1;jsessionid=9A1A7139B2E0E59B89F78430D5440630 23772567

[vms31100-bib-0013] Godfroid, J. , Cloeckaert, A. , Liautard, J. ‐ P. , Kohler, S. , Fretin, D. , Walravens, K. , Garin‐Bastuji, B. , & Letesson, J. J. (2005). From the discovery of the Malta fever's agent to the discovery of a marine mammal reservoir, brucellosis has continuously been a re‐emerging zoonosis. Veterinary Research, 36(3), 313–326. 10.1051/vetres:2005003 15845228

[vms31100-bib-0014] Godfroid, J. , & Käsbohrer, A. (2002). Brucellosis in the European Union and Norway at the turn of the twenty‐first century. Veterinary Microbiology, 90(1), 135–145. 10.1016/S0378-1135(02)00217-1 12414139

[vms31100-bib-0015] Hegazy, Y. , Molina‐Flores, B. , Shafik, H. , Ridler, A. , & Guitian, F. (2011). Ruminant brucellosis in upper Egypt (2005–2008). Preventive Veterinary Medicine, 101(3), 173–181. 10.1016/j.prevetmed.2011.05.007 21684026

[vms31100-bib-0016] Junaidu, A. , Oboegbulem, S. , & Salihu, M. (2010). Seroprevalence of brucellosis in prison farm in Sokoto, Nigeria. Asian Journal of Epidemiology, 3(2), 107–111. 10.3923/aje.2008.24.28

[vms31100-bib-0017] Kadohira, M. , McDermott, J. , Shoukri, M. , & Kyule, M. (1997). Variations in the prevalence of antibody to *Brucella* infection in cattle by farm, area and district in Kenya. Epidemiology and Infection, 118(01), 35–41. 10.1017/S0950268896007005 9042033 PMC2808770

[vms31100-bib-0018] Kubuafor, D. K. , Awumbila, B. , & Akanmori, B. D. (2000). Seroprevalence of brucellosis in cattle and humans in the Akwapim‐South district of Ghana: Public health implications. Acta Tropica, 76(1), 45–48. 10.1016/S0001-706X(00)00088-7 10913765

[vms31100-bib-0019] Logonda, P. , Otshudiandjeka, J. , & Pato, P. (2022). Seroprevalence and risk behaviors of bovine brucellosis in the south‐western prefecture of Haho, Togo, January to April 2020. International Journal of Infectious Diseases, 116, S73. 10.1016/j.ijid.2021.12.172

[vms31100-bib-0020] Magona, J. , Walubengo, J. , Galiwango, T. , & Etoori, A. (2009). Seroprevalence and potential risk of bovine brucellosis in zerograzing and pastoral dairy systems in Uganda. Tropical Animal Health and Production, 41(8), 1765–1771. 10.1007/s11250-009-9375-y 19468854

[vms31100-bib-0021] Mai, H. M. , Irons, P. C. , Kabir, J. , & Thompson, P. N. (2012). A large seroprevalence survey of brucellosis in cattle herds under diverse production systems in northern Nigeria. BMC Veterinary Research, 8(1), 144. 10.1186/1746-6148-8-144 22920578 PMC3482151

[vms31100-bib-0022] Makita, K. , Fèvre, E. M. , Waiswa, C. , Eisler, M. C. , Thrusfield, M. , & Welburn, S. C. (2011). Herd prevalence of bovine brucellosis and analysis of risk factors in cattle in urban and peri‐urban areas of the Kampala economic zone, Uganda. BMC Veterinary Research, 7, 60. 10.1186/1746-6148-7-60 22004574 PMC3212899

[vms31100-bib-0023] Megersa, B. , Biffa, D. , Niguse, F. , Rufael, T. , Asmare, K. , & Skjerve, E. (2011). Cattle brucellosis in traditional livestock husbandry practice in Southern and Eastern Ethiopia, and its zoonotic implication. Acta Veterinaria Scandinavica, 53(1), 1–8. 10.1186/1751-0147-53-24 21473760 PMC3083371

[vms31100-bib-0024] Minas, A. (2006). Control and eradication of brucellosis in small ruminants. Small Ruminant Research, 62(1), 101–107. 10.1016/j.smallrumres.2005.07.031

[vms31100-bib-0025] Moreno, E. (2002). Brucellosis in Central America. Veterinary Microbiology, 90(1), 31–38. 10.1016/S0378-1135(02)00242-0 12414131

[vms31100-bib-0026] Muma, J. , Syakalima, M. , Munyeme, M. , Zulu, V. , Simuunza, M. , & Kurata, M. (2013). Bovine Tuberculosis and Brucellosis in Traditionally Managed Livestock in Selected Districts of Southern Province of Zambia. Veterinary Medicine International, 730367, 10.1155/2013/730367 23862096 PMC3703422

[vms31100-bib-0027] Ocholi, R. , Kwaga, J. , Ajogi, I. , & Bale, J. (2004). Phenotypic characterization of *Brucella* strains isolated from livestock in Nigeria. Veterinary Microbiology, 103(1), 47–53. 10.1016/j.vetmic.2004.06.012 15381265

[vms31100-bib-0029] Omer, M. , Skjerve, E. , Woldehiwet, Z. , & Holstad, G. (2000). Risk factors for *Brucella* spp. infection in dairy cattle farms in Asmara, State of Eritrea. Preventive Veterinary Medicine, 46(4), 257–265. 10.1016/S0167-5877(00)00152-5 10960712

[vms31100-bib-0030] Pandeya, Y. , Joshi, D. , Dhakal, S. , Ghimire, L. , Mahato, B. , Chaulagain, S. , Satyal, R. , & Sah, S. (2013). Seroprevalence of brucellosis in different animal species of Kailali district, Nepal. International Journal of Infection and Microbiology, 2(1), 22–25. 10.3126/ijim.v2i1.8005

[vms31100-bib-0031] Pappas, G. , Papadimitriou, P. , Akritidis, N. , Christou, L. , & Tsianos, E. V. (2006). The new global map of human brucellosis. The Lancet Infectious Diseases, 6(2), 91–99. 10.1016/S1473-3099(06)70382-6 16439329

[vms31100-bib-0032] Pappas, G. , Markoula, S. , Seitaridis, S. , Akritidis, N. , & Tsianos, E. (2005). Brucellosis as a cause of carpal tunnel syndrome. Annals of the Rheumatic Diseases, 64(5), 792. 10.1136/ard.2004.028944 15834067 PMC1755493

[vms31100-bib-0033] Patel, M. , Patel, P. , Prajapati, M. , Kanani, A. , Tyagi, K. , & Fulsoundar, A. (2014). Prevalence and risk factor's analysis of bovine brucellosis in peri‐urban areas under intensive system of production in Gujarat, India. Veterinary World, 7(7), 509–516. 10.14202/vetworld.2014.509-516

[vms31100-bib-0034] Poester, F. P. , Gonçalves, V. t. S. P. , & Lage, A. P. (2002). Brucellosis in Brazil. Veterinary Microbiology, 90(1), 55–62. 10.1128/2FIAI.01855-06 12414134

[vms31100-bib-0035] Preis, I. S. , Moura, A. , de Souza, P. G. , Filho, P. M. S. , & Fonseca Júnior, A. A. (2019). Comparison of six methods of DNA extraction for the diagnosis of bovine brucellosis by real‐time PCR. Archives of Microbiology, 201(8), 1025–1028. 10.1007/s00203-019-01675-3 31101955

[vms31100-bib-0036] Radostits, O. M. , Gay, C. C. , Hinchcliff, K. W. , & Constable, P. D. (2006). Veterinary Medicine: A textbook of the diseases of cattle, horses, sheep, pigs and goats. Elsevier Health Sciences.

[vms31100-bib-0037] Ragan, V. E. (2002). The animal and plant health inspection service (APHIS) brucellosis eradication program in the United States. Veterinary Microbiology, 90(1), 11–18. 10.1016/S0378-1135(02)00240-7 12414129

[vms31100-bib-0038] Rahman, A. (2015). Epidemiology of brucellosis in humans and domestic ruminants in Bangladesh. PhD Thesis, Institue of Tropical Medicine. Retrieved from http://hdl.handle.net/2268/178980

[vms31100-bib-1038] Rahman, M. S. , Uddin, M. J. , Park, J. H. , Chae, J. S. , Rahman, M. B. , & Islam, M. A. (2006). A short history of brucellosis: special emphasis in Bangladesh. Bangladesh Journal of Veterinary Medicine, 4(1), 1–6. 10.3329/bjvm.v4i1.1517

[vms31100-bib-0039] Rahman, A. A. , Saegerman, C. , Berkvens, D. , Fretin, D. , Gani, M. O. , Ershaduzzaman, M. , Ahmed, M. U. , & Emmanuel, A. (2013). Bayesian estimation of true prevalence, sensitivity and specificity of indirect ELISA, Rose Bengal Test and Slow Agglutination Test for the diagnosis of brucellosis in sheep and goats in Bangladesh. Preventive Veterinary Medicine, 110(2), 242–252. 23276401 10.1016/j.prevetmed.2012.11.029

[vms31100-bib-0040] Rahman, M. , Faruk, M. , Her, M. , Kim, J. , Kang, S. , & Jung, S. (2011). Prevalence of brucellosis in ruminants in Bangladesh. Veterinarni Medicina, 56(8), 379–385. 10.17221/1555-VETMED

[vms31100-bib-0041] Rahman, S. , Huque, F. , Ahasan, S. , & Song, H. J. (2010). Indirect enzyme linked immunosorbent assay for the diagnosis of brucellosis in cattle. Korean Journal of Veterinary Service, 33(2), 113–119.

[vms31100-bib-0042] Scacchia, M. , Di Provvido, A. , Ippoliti, C. , Kefle, U. , Sebhatu, T. T. , D'Angelo, A. , & De Massis, F. (2013). Prevalence of brucellosis in dairy cattle from the main dairy farming regions of Eritrea. Onderstepoort Journal of Veterinary Research, 80(1), 448. 10.4102/ojvr.v80i1.448 23718833

[vms31100-bib-0043] Shafee, M. , Rabbani, M. , Sheikh, A. A. , & Razzaq, A. (2011). Prevalence of bovine brucellosis in organized dairy farms, using milk ELISA, in Quetta City, Balochistan, Pakistan. Veterinary Medicine International, 2011, 358950. 10.4061/2011/358950 21331157 PMC3034933

[vms31100-bib-0044] Thursfield, M. (2005). Veterinary Epidemiology. (3rd ed.) (P233). London. Wiley‐Blackwell.

[vms31100-bib-0045] von Bargen, K. , Gorvel, J.‐P. , & Salcedo, S. P. (2012). Internal affairs: Investigating the *Brucella* intracellular lifestyle. FEMS Microbiology Reviews, 36(3), 533–562. 10.1111/j.1574-6976.2012.00334 22373010

[vms31100-bib-0046] ZareBidaki, M. , Allahyari, E. , Zeinali, T. , & Asgharzadeh, M. (2022). Occurrence and risk factors of brucellosis among domestic animals: An artificial neural network approach. Journal of Tropical Animal Health and Production, 54(1), 62. 10.1007/s11250-022-03076-z 35037143

